# Effect of polymerization mode on shrinkage kinetics and degree of conversion of dual-curing bulk-fill resin composites

**DOI:** 10.1007/s00784-023-04928-0

**Published:** 2023-03-04

**Authors:** Phoebe Burrer, Matej Par, Leo Fürer, Michelle Stübi, Danijela Marovic, Zrinka Tarle, Thomas Attin, Tobias T. Tauböck

**Affiliations:** 1 Department of Conservative and Preventive Dentistry, Center for Dental Medicine, University of Zurich, Plattenstrasse 11, 8032 Zurich, Switzerland; 2 Department of Endodontics and Restorative Dentistry, School of Dental Medicine, University of Zagreb, Gunduliceva 5, 10000 Zagreb, Croatia

**Keywords:** Bulk-fill composites, Dual-curing, Polymerization shrinkage behavior, Degree of conversion

## Abstract

**Objectives:**

To assess the behavior of dual-cure and conventional bulk-fill composite materials on real-time linear shrinkage, shrinkage stress, and degree of conversion.

**Materials and methods:**

Two dual-cure bulk-fill materials (Cention, Ivoclar Vivadent (with ion-releasing properties) and Fill-Up!, Coltene) and two conventional bulk-fill composites (Tetric PowerFill, Ivoclar Vivadent; SDR flow + , Dentsply Sirona) were compared to conventional reference materials (Ceram.x Spectra ST (HV), Dentsply Sirona; X-flow; Dentsply Sirona). Light curing was performed for 20 s, or specimens were left to self-cure only. Linear shrinkage, shrinkage stress, and degree of conversion were measured in real time for 4 h (*n* = 8 per group), and kinetic parameters were determined for shrinkage stress and degree of conversion. Data were statistically analyzed by ANOVA followed by post hoc tests (*α* = 0.05). Pearson’s analysis was used for correlating linear shrinkage and shrinkage force.

**Results:**

Significantly higher linear shrinkage and shrinkage stress were found for the low-viscosity materials compared to the high-viscosity materials. No significant difference in degree of conversion was revealed between the polymerization modes of the dual-cure bulk-fill composite Fill-Up!, but the time to achieve maximum polymerization rate was significantly longer for the self-cure mode. Significant differences in degree of conversion were however found between the polymerization modes of the ion-releasing bulk-fill material Cention, which also exhibited the significantly slowest polymerization rate of all materials when chemically cured.

**Conclusions:**

While some of the parameters tested were found to be consistent across all materials studied, heterogeneity increased for others.

**Clinical relevance:**

With the introduction of new classes of composite materials, predicting the effects of individual parameters on final clinically relevant properties becomes more difficult.

## Introduction

In recent years, modern conservative dentistry has been revolutionized by several innovative developments aiming at the simplification of the restoration process and improving the interaction of the restoration material with the adjacent dental hard tissues. The use of modern bulk-fill composite materials designed for layering up to 4–5 mm or more has rendered the restoration process more time-efficient and less prone to application flaws, while maintaining adequate mechanical and physical properties [1–5]. Bulk-fill composites can be classified into low-viscosity (flowable) and high-viscosity (sculptable) material types based on differences in their rheological properties and application techniques [3]. Moreover, the restoration process can be further simplified by using composite materials with dual-curing features, allowing both a self-cure as well as light-cure polymerization [6–9]. Another recent innovative development is the introduction of “smart” composites, describing resin-based, ion-releasing restorative materials with antibacterial properties also promoting remineralization at the bonded interface [10]. Those commercially available or experimental materials incorporated with various amounts of inorganic fillers such as bioactive glass particles have the promising ability to interact with the surrounding tooth structure, in contrast to conventional restorative materials [7, 11–15].

Thus, in combination with dual-curing and bulk-filling abilities, promising materials have been developed. The recently launched dual-cure restorative material Cention, Cention N, or Cention Forte (Ivoclar Vivadent, Schaan, Liechtenstein; name and approval depending on geographic region) with alkaline fillers can be applied in a single layer and used in self-cure or light-cure polymerization mode and is specifically aimed for economical upcoming countries. It releases hydroxide ions compensating for a pH value that has been lowered due to bacterial colonization, while the remineralization process is further supported by release of fluoride, phosphate, and calcium ions [16–21]. Another recent material that has been launched to the dental market is the dual-cure bulk-fill material Fill-Up! (Coltene/Whaledent AG, Altstätten, Switzerland) containing antibacterial zinc oxide particles [22, 23]. Fill-Up! is a microhybrid, two-component composite that can be applied in any layer thickness, while it is promoted to produce minimal shrinkage stress and unlimited curing depth. Both light-curing mode and self-curing mode are provided [22].

To date, the question of how different polymerization modes of dual-curing bulk-fill materials affect mechanical and chemical parameters cannot be answered unambiguously based on the literature available. These parameters can be assessed among others by measuring degree of conversion and polymerization shrinkage of the composite materials. The latter creates stresses at the tooth-restoration interface during curing, and if the shrinkage forces exceed the bond strength between the tooth and restoration material, localized debonding might occur [24]. Therefore, the aim of the present study was to assess the influence of polymerization mode on shrinkage stress formation, polymerization shrinkage, and polymerization kinetics of two dual-curing bulk-fill materials compared to conventional bulk-fill and non-bulk-fill resin composites. The tested null hypotheses were that (i) there is no difference in linear polymerization shrinkage, shrinkage stress development, and polymerization kinetics between the dual-cure bulk-fill, conventional bulk-fill, and the reference composites; and (ii) that there is no difference in the aforementioned properties between the self-cure and light-cure polymerization mode of the dual-cure bulk-fill composite materials.

## Materials and methods

### Composite materials

The manufacturers’ information, classification, and composition of the six commercial composite materials used in the present study are depicted in Table 1. Four of the tested materials were bulk-fill composites (Fill-Up!, Coltene; Cention, Ivoclar Vivadent; Tetric PowerFill, Ivoclar Vivadent; and SDR flow + , Dentsply Sirona), of which Fill-Up and Cention can be additionally regarded as dual-curing composites, with Cention also revealing ion-releasing properties. Two conventional resin composites, one sculptable (Ceram.x Spectra ST (HV), Dentsply Sirona) and one flowable (X-flow, Dentsply Sirona) were used as references. When applied, light curing was performed for 20 s using an LED light-curing unit (Bluephase PowerCure, Ivoclar Vivadent, Schaan, Liechtenstein) in high-intensity mode. The output irradiance of 1340 mW/cm^2^ was verified at regular intervals with a calibrated FieldMax II-TO power meter and PM2 thermopile sensor (Coherent, Santa Clara, CA, USA).

**Table 1:** Manufacturers’ information about the composite materials used in the present study

Material type	Special features	Composite viscosity	Material name	Composition	Filler content (wt%/vol%)	Lot no./shade	Manufacturer
Bulk-fill	Dual-cure	Flowable- medium viscosity	Fill-Up!	Matrix: TMPTMA^ 1^, UDMA^ 2^, Bis-GMA^ 3^, TEGDMA^ 4^ Filler: Zinc oxide coated, dental glass, amorphous silica Photo-initiator: Dibenzoyl peroxide, benzoyl peroxide	65/49	J87573/universal	Coltene/ Whaledent, Altstätten, Switzerland
	Dual-cure, ion-releasing	Medium viscosity	Cention (Alkasite)	Matrix: UDMA^ 2^, DCP^ 5^, aromatic-aliphatic-UDMA^ 2^, PEG-400 DMA^ 6^ Filler: Ca-F-silicate glass, Ba-Al–silicate glass, Ca-Ba-Al-fluorosilicate glass, ytterbium trifluoride, isofiller Photo-initiator: Hydroperoxide, Ivocerin, acyl phosphine oxide	78/58	Z005MC/A2	Ivoclar Vivadent, Schaan, Liechtenstein
	Control	Sculptable	Tetric PowerFill	Matrix: Bis-GMA^ 3^, Bis-EMA^ 7^, UDMA^ 2^, propoxylated bisphenol-A dimethacrylate, DCP^ 5^ Filler: Barium glass, ytterbium fluoride, mixed oxide, copolymers, additives, stabilizers, pigments Photo-initiator: CQ ^8^, amine, Ivocerin, Lucirin TPO^ 9^	76–77/53–54	X56571/^IV^A	Ivoclar Vivadent, Schaan, Liechtenstein
	Control	Flowable	SDR flow +	Matrix: Proprietary modified urethane dimethacrylate resin, TEGDMA ^4^, polymerizable dimethacrylate resin, polymerizable trimethacrylate resin, BHT ^10^, fluorescent agent, UV stabilizer Filler: Silanated barium-alumino-fluoro-borosilicate glass, silanated strontium-alumino-fluoro-silicate glass, surface treated fume silicas, ytterbium fluoride, synthetic inorganic iron oxide pigments, titanium dioxide Photo-initiator: CQ^ 8^, EDMAB^ 11^	70.5/47.4	00,020,472/universal	Dentsply Sirona, Konstanz, Germany
Conventional	Control	Sculptable	Ceram.x Spectra ST (HV)	Matrix: Methacrylic modified polysiloxane nanoparticles, dimethacrylate resin Filler: Spherical, pre-polymerized SphereTEC fillers (particle size < 0.1 μm), non-agglomerated barium glass, ytterbium fluoride Photo-initiator: CQ ^8^, EDMAB^ 11^	78–80/60–62	2,009,000,146/A2	Dentsply Sirona, Konstanz, Germany
	Control	Flowable	X-flow	Matrix: Multifunctional acrylate resin, difunctional methacrylate resin, DGDMA ^12^, UV stabilizer, BHT^ 10^ Filler: Strontium-alumino-sodium-fluoro-phosphor-silicate glass, highly dispersed silicon dioxide, iron oxide pigments, titanium dioxide Photo-initiator: CQ ^8^, EDMAB^ 11^	60/38	2,003,000,579/A2	Dentsply Sirona, Konstanz, Germany

^1^TMPTMA: trimethylolpropane-trimethacrylate

^2^UDMA: urethane dimethacrylate

^3^Bis-GMA: bisphenol-A-glycidyl-dimethacrylate

^4^TEGDMA: triethylene glycol dimethacrylate

^5^DCP: tricyclodecan-dimethanol dimethacrylate

^6^PEG-400 DMA: polyethylene glycol 400 dimethacrylate

^7^Bis-EMA: ethoxylated bisphenol-A-glycidyl methacrylate

^8^CQ: camphorquinone

^9^TPO: 2,4,6-trimethylbenzoyldiphenylphosphine oxide

^10^BHT: butylated hydroxy toluene

^11^EDMAB: ethyl-4(dimethylamino)benzoate photoaccelerator

^12^DGDMA: diethylene glycol dimethacrylate

### Linear polymerization shrinkage

Linear polymerization shrinkage of all materials was measured with a custom-built linometer adapted from de Gee et al. [25] and already described in detail in the literature [3, 8, 26–29]. In short, a thin aluminum plate (12 × 12 mm; thickness: 0.25 mm) with an attached perpendicular diaphragm was loosely placed in a solid metal frame of the linometer. The lowest part of the vertical diaphragm protruded into a recess of the light barrier of the linometer with an infrared measuring sensor. Standardized amounts of all materials were prepared by filling the sculptable materials into a cylindrical Teflon mold (42 mm^3^) and then carefully transferring on the platelet, while the flowable materials were directly weighed on the aluminum platelet with the corresponding reference weight of 0.083 ± 0.005 g with a precision balance (Sartorius Analytic; Sartorius, Göttingen, Germany) to obtain the standardized volume of 42 mm^3^. In the linometer, the applied material was then pressed to a thickness of 1.5 mm by a glass plate (Menzel-Gläser; ThermoFisher Scientific, Waltham, MA, USA; 25 × 42 × 1 mm). To enhance adhesion, each glass plate was previously sandblasted with 50-µm aluminum oxide powder, thoroughly rinsed and silanized (Monobond Plus, Ivoclar Vivadent). Light polymerization was performed, if applied according to the study protocol, through the glass plate for 20 s under direct contact of the light guide tip. Material specimens intended for self-polymerization were prepared for data recording within 30 s after dispensing from the syringe (Fill-Up!) or capsule (Cention). The vertical movement of the diaphragm triggered by the polymerization shrinkage of the tested materials was registered by the infrared sensor in a temperature-controlled setting of 25 ± 1 °C, simulating intra-oral temperature after placement of rubber dam [30]. Measurements were recorded at a data sampling frequency of 1 Hz and an accuracy of 0.1 µm during 4 h from the start of polymerization. During the 4-h measurements, data were transferred in real time to an attached computer (Macintosh Ilfx; Apple Computer, Cupertino, CA, USA) by means of an analog-to-digital converter and custom-made software. Eight replicate measurements were performed for each experimental group (*n* = 8) and mean values were calculated. Additionally, one measurement per material and polymerization mode was performed for 24 h to ensure that no further increase in linear shrinkage had occurred after 4 h.

### Polymerization shrinkage stress

Real-time measurements of polymerization shrinkage stress were performed using a custom-made stress analyzer also previously described in detail [3, 8, 26–28, 31]. Briefly, a metal cylinder was screwed to a semi-rigid load cell (PM 11-K; Mettler, Greifensee, Switzerland; instrument compliance: 0.4 µm/N). As described for linear shrinkage measurements, a standardized amount of material (42 mm^3^ or 0.083 ± 0.005 g) was placed on the cylinder and pressed by a glass plate (Menzel-Gläser; ThermoFisher Scientific, Waltham, MA, USA; 20 × 26 × 1 mm) to a thickness of 1.5 mm, resulting in a base surface area of 28 mm^2^ and a ratio of bonded-to-unbonded surface (C-factor) of 2.0. Both metal cylinder and glass plate were sandblasted (50 µm Al_2_O_3_) and silanized (Monobond Plus; Ivoclar Vivadent) and checked under a stereomicroscope at 40 × magnification (M3Z; Leica/Wild, Heerbrugg, Switzerland) to ensure that no remnants of aluminum oxide powder were left on the surfaces. Light polymerization was again performed through the glass plate under direct contact for 20 s and the resulting shrinkage forces were recorded by means of the load cell at a sampling frequency of 1 Hz in a controlled chamber temperature of 25 ± 1 °C. Specimens designated for self-polymerization were prepared within 30 s after dispensing and the data was recorded for 4 h. Recorded data were transferred in real time to the attached computer (Macintosh Ilfx; Apple Computer) via the analog-to-digital converter and custom-made software. Eight replicate measurements were performed for each experimental group (*n* = 8) and shrinkage stress (MPa) was calculated by dividing the obtained shrinkage force data by the bonded surface area (N/mm^2^). One measurement per material and polymerization mode was additionally performed for 24 h to ensure that no further increase in shrinkage stress had occurred after 4 h. Shrinkage stress curves were then plotted as a function of time and the first derivatives of theses curves were calculated to obtain shrinkage stress rate so that the kinetic parameter maximum stress rate (*R*_max_) and time to achieve maximum shrinkage stress rate (*t*_Rmax_) could be determined.

### Real-time degree of conversion and polymerization kinetics

Degree of conversion (DC) (*n* = 8) was measured in real time using Fourier-transform infrared (FTIR) spectrometer (Nicolet iS50, Thermo Fisher, Madison, WI, USA). DC was assessed at the bottom surfaces of the 1.5-mm-thick specimens (42 mm^3^) during 4 h, corresponding to measurements of linear shrinkage and shrinkage stress of the present study. A thickness of 1.5 mm was also chosen for DC measurements of bulk-fill materials to guarantee standardization. The specimens were covered with polyethylene terephthalate foils and light-cured for 20 s or left in dark for self-curing.

FTIR spectra were recorded in real time (2 spectra per second), using 4 scans and a spectral resolution of 8 cm^−1^. Spectra of the uncured dual-cure materials were obtained by starting the FTIR measurements immediately after dispensing the mixed material on the ATR crystal and successively recording 4 spectra at a rate of 2 spectra/s, resulting in a total measurement time of 2 s. An average spectrum was calculated from these spectra and used to represent the uncured state of the composite material, since no measurable change in the intensity of the spectral band at 1638 cm^−1^ was observed. DC calculation was performed by comparing the peak heights of the aliphatic C = C spectral bands at 1638 cm^−1^, and the reference (internal standard) bands between the polymerized and unpolymerized specimens according to Eq. (1) [26]:

1 $$\mathrm{DC}\;\left(\%\right)=\left[1-\left.\frac{{\lbrack{\mathrm{Abs}\;(1638\mathrm{cm}}^{-1}{)\;/}\;{\mathrm{Abs}\;(\mathrm{reference}}{)\rbrack}}_{\mathrm{cured}}}{{\lbrack{\mathrm{Abs}\;(1638\mathrm{cm}}^{-1}{)\;/}\;{\mathrm{Abs}\;(\mathrm{reference}}{)\rbrack}}_{\mathrm{uncured}}}\right]\right.\times100$$

The spectral band at 1608 cm^−1^ (aromatic C···C) was used as a reference band for Tetric PowerFill, SDR flow + , and Ceram.x Spectra ST (HV), while the spectral band at 1454 cm^−1^ (C-H stretching) was used for Fill-Up!, Cention, and X-flow. From the plots of DC as a function of time, first derivatives were calculated to assess the real-time reaction rate. By plotting the reaction rate as a function of time, maximum reaction rate (*R*_DCmax_) and time to reach maximum reaction rate (*t*_DCmax_) were determined. The DC values measured at the end of the 4-h period (DC_4h_) were additionally evaluated.

### Statistical analysis

After testing for normality and homogeneity of variances using Shapiro Wilk’s and Levene’s test, data were statistically analyzed between materials and curing modes using one-way analysis of variance (ANOVA) followed by pairwise HSD post hoc tests corrected for multiple comparisons according to Tukey to detect differences in the outcome variables linear shrinkage, shrinkage stress, and degree of conversion. Shrinkage stress and degree of conversion kinetics data were analyzed using Welch ANOVA, followed by Games-Howell post hoc tests across all combinations of material and curing mode. Pearson’s correlation analysis was used to examine the relationship between linear shrinkage and shrinkage force. All statistical analyses were performed using SPSS version 27 (IBM Corp. Armonk, NY, USA). The overall level of significance was set to *α* = 0.05.

## Results

**Fig. 1:**
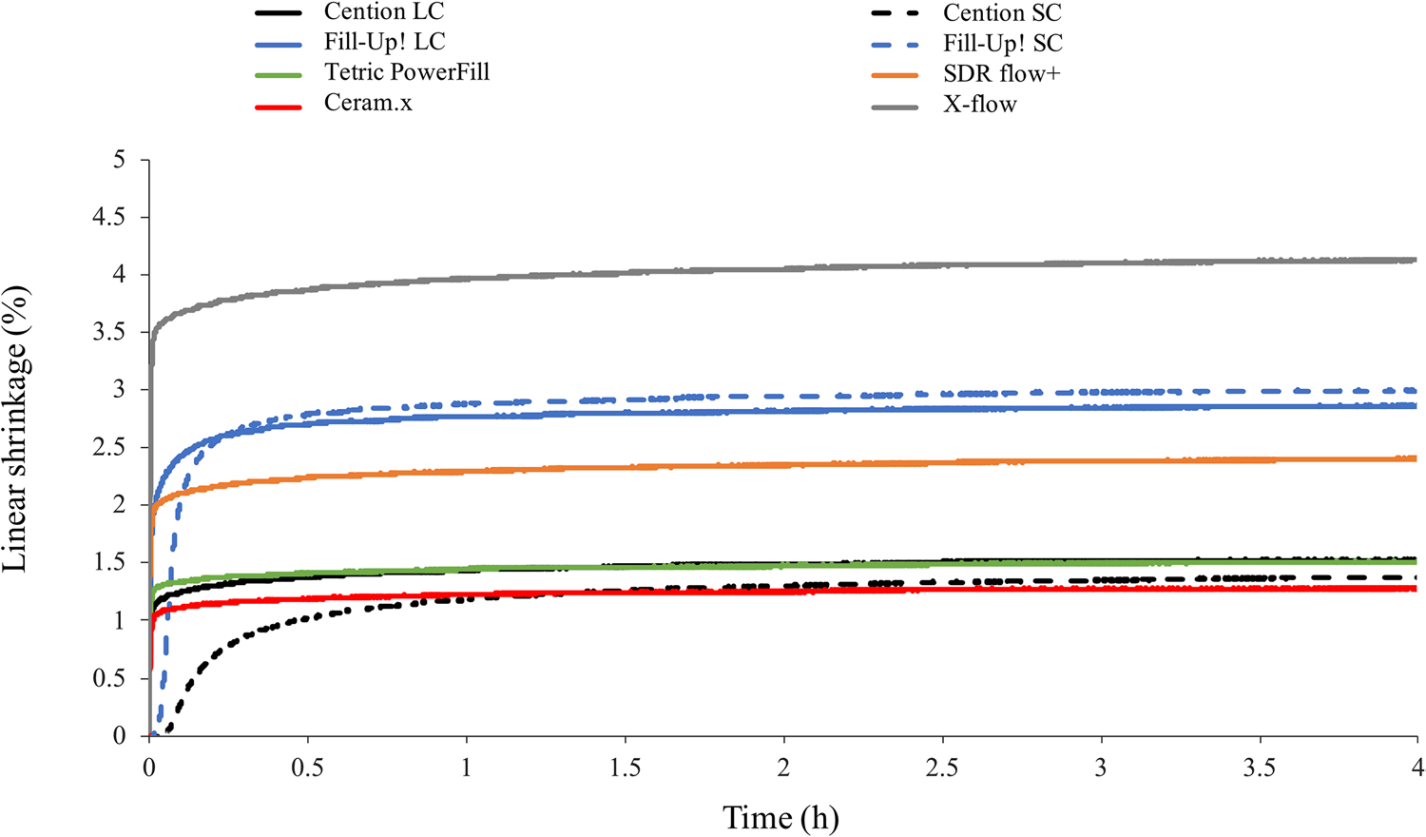
Time-dependent mean linear shrinkage curves of all tested materials and polymerization modes. SC: self-cured; LC: light-cured

**Fig. 2:**
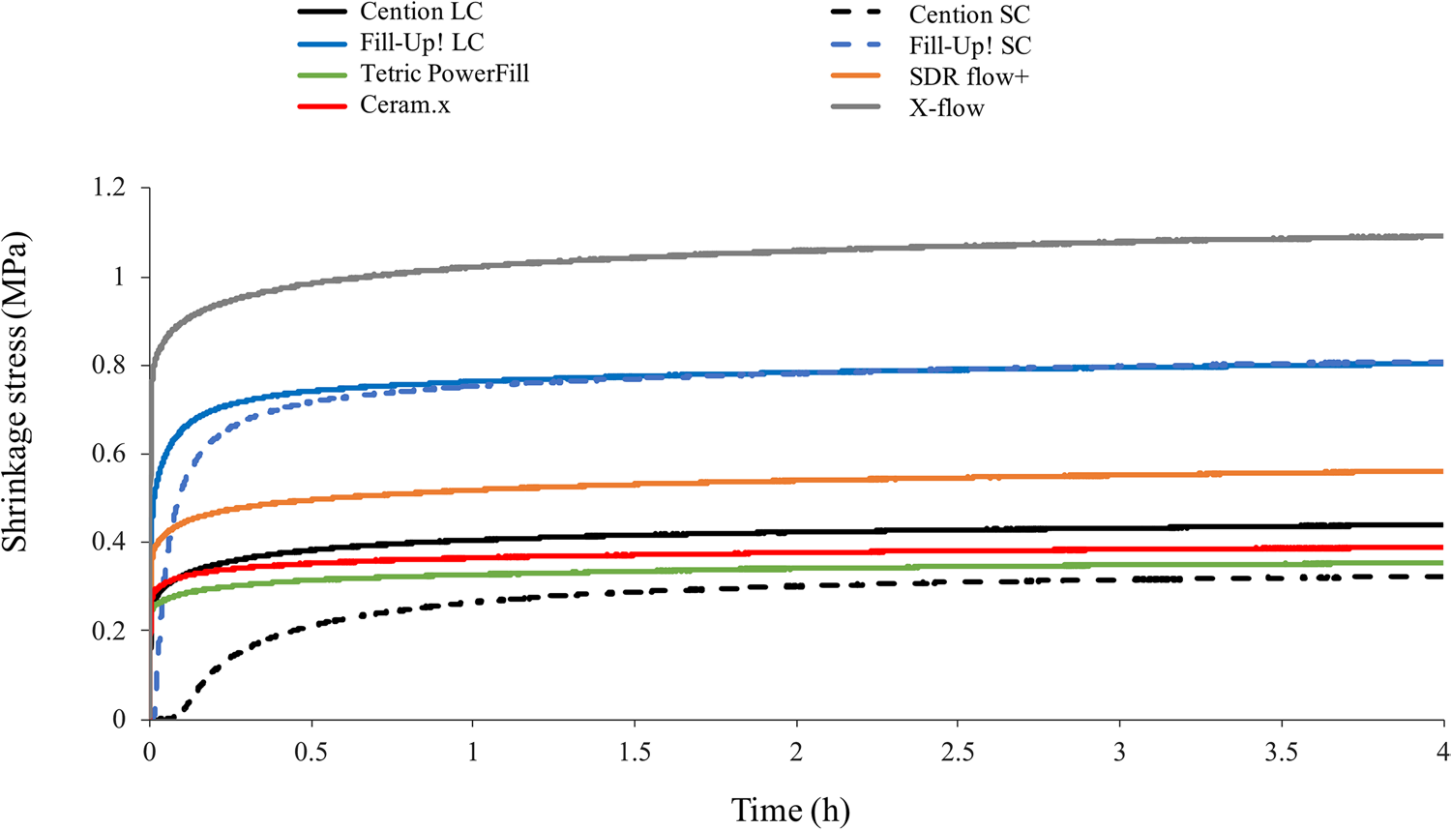
Time-dependent mean shrinkage stress curves of all tested materials and polymerization modes. SC: self-cured; LC: light-cured

**Fig. 3:**
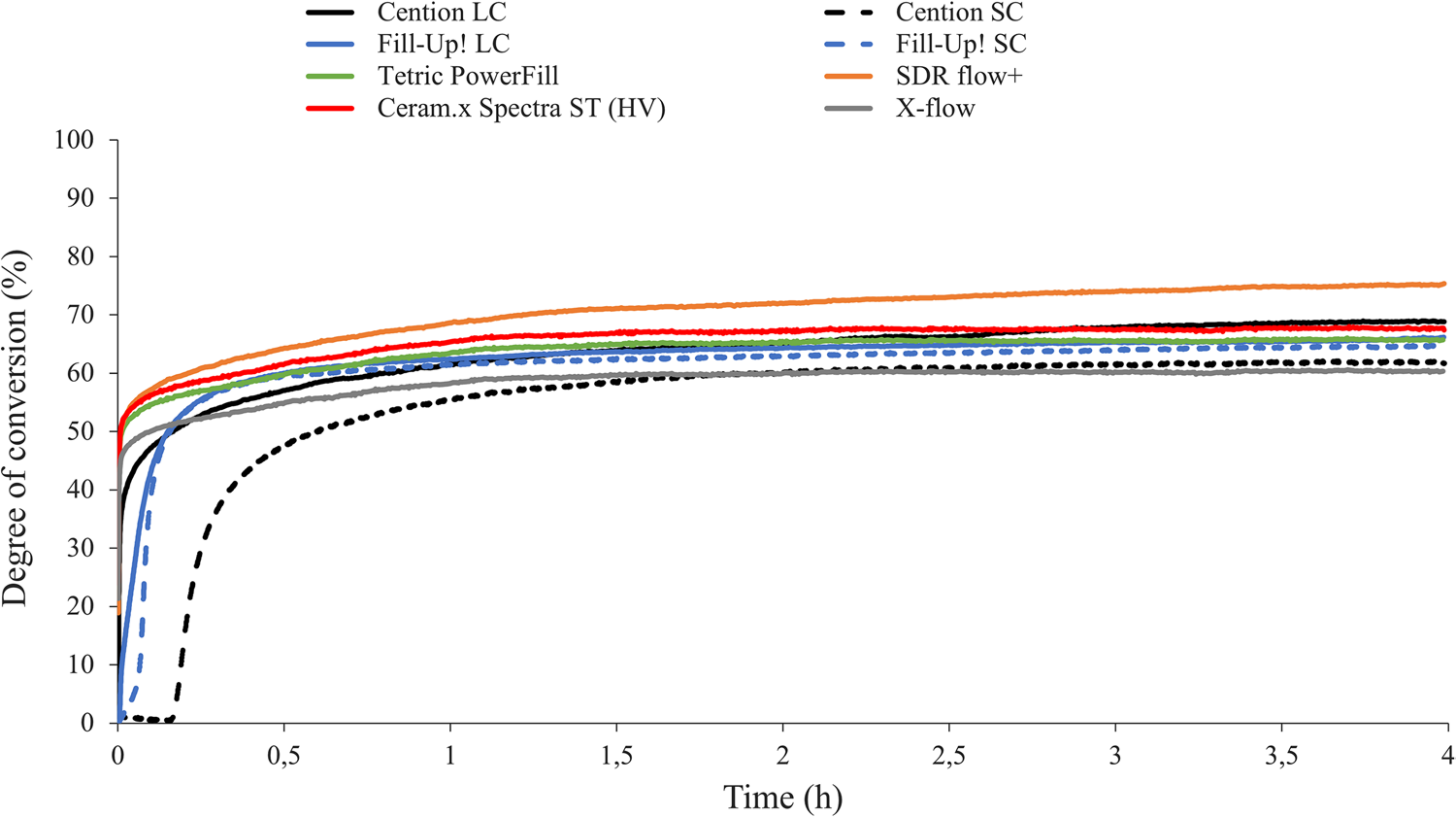
Time-dependent mean degree of conversion curves of all tested materials and polymerization modes. SC: self-cured; LC: light-cured

Figures 1, 2, and 3 illustrate the real-time development of linear shrinkage, shrinkage stress, and degree of conversion, respectively. The nominal values of linear shrinkage, shrinkage stress, and DC registered at the end of the 4-h observation period, and the calculated values of maximum shrinkage stress rate (*R*_max_), time to achieve the maximum shrinkage stress rate (*t*_Rmax_), the maximum polymerization rate (*R*_*DC*,max_), and the time to achieve the maximum polymerization rate (*t*_R,DC,max_) are presented in Table 2.

**Table 2:** Mean values (± standard deviation) of the parameters linear shrinkage, shrinkage stress, maximum shrinkage stress rate (*R*_max_), time to achieve maximum stress rate (*t*_Rmax_), degree of conversion, maximum polymerization rate (*R*_*DC*,max_), and time to achieve maximum polymerization rate (*t*_R,DC,max_). Within each column, same capital letters indicate no significant difference at a significance level of 0.05. SC: self-cured; LC: light-cured

Material	Linear shrinkage (%)	Shrinkage stress (MPa)	*R*_max_ (MPa/s)	*t*_Rmax_ (s)	Degree of conversion (%)	*R*_DC,max_ (%/s)	*t*_R,DC,max_ (s)
Fill-Up! (SC)	2.99 (0.18) B	0.81 (0.11) B	0.0029 (0.001) D	110.13 (3.94) B	64.70 (1.17) B	0.30 (0.05) D	258.24 (47.08) B
Fill-Up! (LC)	2.86 (0.18) B	0.80 (0.04) B	0.0323 (0.005) C	8.54 (1.56) C	65.78 (1.90) B	0.39 (0.05) D	26.64 (5.61) C
Cention (SC)	1.37 (0.06) DE	0.32 (0.03) E	0.0003 (0.000) E	433.63 (58.90) A	61.38 (2.23) C	0.15 (0.02) E	642.00 (102.09) A
Cention (LC)	1.52 (0.06) D	0.44 (0.06) D	0.0263 (0.005) C	2.61 (1.12) EF	68.08 (1.67) B	5.70 (1.82) B	3.04 (0.58) E
Tetric PowerFill (LC)	1.51 (0.06) D	0.35 (0.05) E	0.0383 (0.010) BC	1.55 (0.96) F	66.14 (1.62) B	13.70 (1.00) A	2.34 (0.17) F
SDR flow + (LC)	2.40 (0.09) C	0.56 (0.02) C	0.0421 (0.004) B	3.61 (0.81) DE	75.18 (2.51) A	12.60 (1.00) A	2.60 (0.35) F
Ceram.x Spectra ST (HV) (LC)	1.28 (0.08) E	0.39 (0.03) DE	0.0265 (0.003) C	3.68 (1.66) DEF	67.20 (1.48) B	7.97 (0.57) B	3.95 (0.40) E
X-flow (LC)	4.12 (0.16) A	1.09 (0.06) A	0.0725 (0.009) A	4.81 (1.19) D	59.90 (1.40) C	4.01 (0.37) C	6.73 (0.54) D

The greatest changes in all materials in linear shrinkage (Fig. 1) occurred within about the first half hour of polymerization. At the end of the 4-h measurement period, only very small changes in shrinkage stress were observed (asymptotic behavior). Among all materials under investigation, significantly higher linear shrinkage was found for the low-viscosity materials (Fill-Up!, SDR flow + , and X-flow) compared to the high-viscosity materials.

As well as for linear shrinkage, for shrinkage stress (Fig. 2), the greatest changes of all materials under investigation were observed within the first half hour of polymerization, again reaching asymptotic behavior. The significantly highest shrinkage stress was observed for X-flow, which also reached the highest maximum stress rate (*R*_max_). For Cention, self-curing led to significantly lower shrinkage stress values compared to light-curing (*p* < 0.001). Self-curing of both Cention and Fill-Up! resulted in significantly lower maximum shrinkage stress rates, but significantly higher times to achieve maximum shrinkage stress rates than light-curing. Additionally, the self-cure polymerization mode of Cention showed the significantly lowest maximum shrinkage stress reaction rate of all materials, while it needed the significantly longest time (more than 7 min) to achieve maximum shrinkage stress rate (*p* < 0.001).

The significantly highest DC of all tested materials (Fig. 3) was attained by SDR flow + , amounting to more than 75%. The lowest DC values were reached by X-flow and the self-cure polymerization mode of Cention, while the latter was also significantly lower than its corresponding light-cure polymerization mode. Regarding polymerization kinetics, Cention SC showed the significantly slowest maximum reaction rate of all tested materials and the significantly longest time to achieve maximum polymerization rate of more than 10 min. Fill-Up! required significantly longer time to reach maximum reaction rate (*p* < 0.001) when self-cured than when light-cured. Both conventional bulk-fill materials Tetric PowerFill and SDR flow + revealed the significantly highest polymerization rate, while showing lowest times to reach their maximum polymerization rate.

**Fig. 4:**
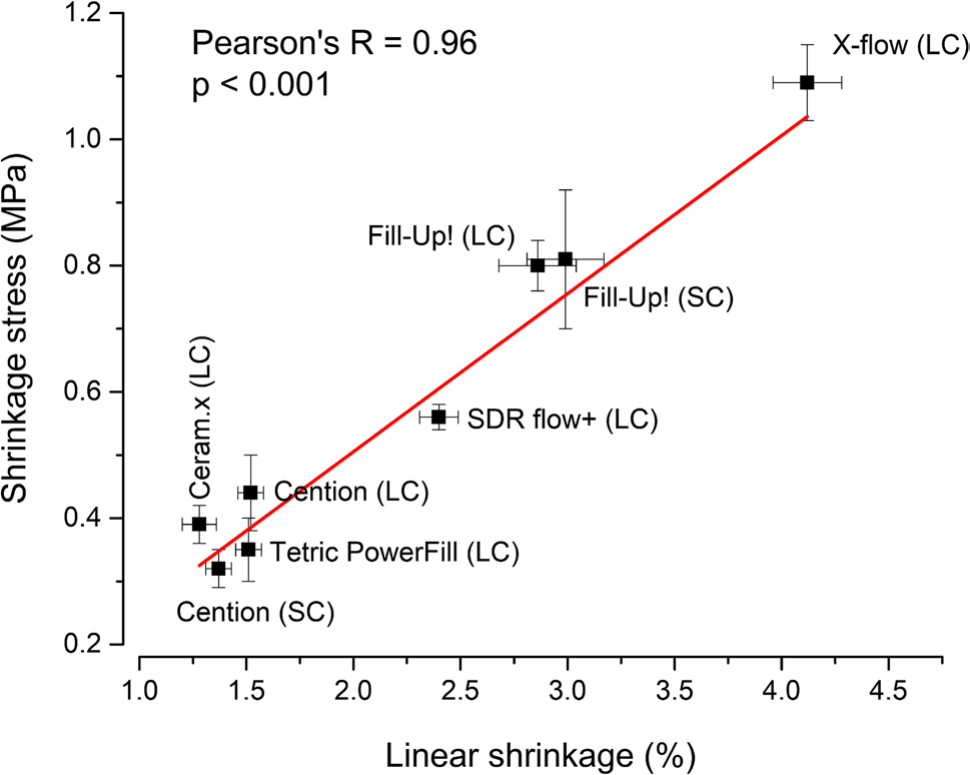
Pearson’s correlation plot of linear shrinkage and shrinkage stress (mean values ± SD). SC: self-cured; LC: light-cured

In Fig. 4, shrinkage stress is plotted as a function of linear shrinkage. A statistically significant correlation was identified with *R* = 0.96.

## Discussion

The combination of the innovative composite features of bulk-filling, dual-curing, and ion-release is of great interest to clinical users, as these materials do not only offer a simplification in handling while exerting a positive effect on the interface between restoration and tooth, but also promise appropriate chemical and physical material properties [1, 4, 19, 32–34]. To enable comparison of the results of the tested bulk-fill composite materials to conventional composites and to maintain a consistent C-factor, a specimen thickness of 1.5 mm was chosen for all measurements. A long-term observation period of 4 h was chosen for all materials, after 24-h measurements had previously been performed to ensure that no further significant increase was observed beyond 4 h.

Results of the present study showed significant differences in all tested parameters between the different flowable dual-cure and conventional bulk-fill materials compared to the conventional flowable composite, which leads to the rejection of the first null hypothesis. Overall, results showed that flowable composite materials had a higher linear shrinkage and exhibited higher shrinkage stress than their sculptable counterparts, which is in accordance with other studies [31, 35]. Additionally, within the low-viscosity materials, bulk-fill composites showed significantly lower shrinkage forces than the conventional flowable, an observation also made previously [36]. The composition of the composite material has a significant impact on the resulting shrinkage stress [37] and might explain the differences in the shrinkage stress development between the different bulk-fill composites. Hereby, in both polymerization modes, the dual-cure bulk-fill material Fill-Up! showed second highest shrinkage stress values of all materials. Fill-Up! consists of two components that are mixed together during extrusion, a process which is enabled by the lower material viscosity at the cost of higher volumetric shrinkage. Mostly, low-viscosity bulk-fill composites are less filled and have comparatively large particles, which reflects on their shrinkage properties, as could be observed in the present study. However, it must be pointed out that the flowable bulk-fill material SDR flow + generated the significantly lowest polymerization shrinkage stress of all flowable materials. This might be attributed on the one hand to the larger size of the SDR resin monomers compared to conventional resin systems with a molecular weight of 849 g/mol for SDR resin compared to 513 g/mol for Bis-GMA [38] and on the other hand to the contained proprietary modified UDMA that can delay the development of shrinkage stresses [39].

A direct correlation between the extent of contraction forces generated and the rate and degree of conversion has been shown by several studies, with a high degree of conversion required for the performance of the material in terms of mechanical properties [7, 26, 35, 40]. In general, an increased degree of conversion of bulk-fill resin composites compared to conventional composites might be attributed to their higher translucency facilitating light transmittance, reduced filler load, or larger filler particles reducing light scattering and reflection [41–44]. Our results however only reflect these observations regarding the low-viscosity materials, indicating that additionally other important factors affected the degree of conversion, such as the initial viscosity, which is dictated by the lower filler content of the low-viscosity materials, and the flexibility of the monomer structure [45]. In the present study, the significantly highest DC of all tested materials was found for the flowable bulk-fill composite SDR flow + , which might be attributed to its modified UDMA that retards polymerization and delays immobilization of the resin, allowing it to reach higher conversion values. Interestingly, all other bulk-fill materials (except Cention SC) and Ceram.x Spectra ST (HV) achieved similar DC values to each other, regardless of their viscosity and filler content. DC values of all materials ranged between 60 and 75%, which agrees with other studies [46, 47]. Moreover, DC results for the bulk-fill composite Tetric PowerFill and the conventional resin composite Ceram.x Spectra ST (HV) of the present study were even higher compared to previous studies [13, 26, 48], which might be mainly attributed to the different study parameters, such as light curing times and intensities. When comparing the maximum polymerization rate for the composite specially designed for high-intensity light-curing (Tetric PowerFill) to current literature [48], also comparably higher values were attained in the present study. For interpreting both DC and shrinkage results, the photo-initiator system of the tested materials additionally plays a significant role. Both Cention and Tetric Power Fill contain an additional Germanium-based initiator Ivocerin, which exhibits higher light reactivity than camphorquinone. Due to the improved light transmission of these bulk-fill materials, the reactivity for light-activated polymerization can be increased [42, 49]. This consideration is also supported by the results of the present study, showing for the Ivocerin-containing materials a good polymerization behavior comparable to other studies [14, 50, 51].

Furthermore, results of the present study revealed significant differences between the self-cure and light-cure polymerization mode of the dual-cure bulk-fill material Cention. Thus, the second null hypothesis had to be rejected. In general, the process of composite self-curing is activated by chemical initiators with a slow initiation rate [47], leading to less shrinkage stress for self-cured than for light-cured composite materials. In the present study, Cention SC exhibited the significantly lowest DC among all dual-cure bulk-fill groups including its light-cured counterpart, and showed the significantly lowest shrinkage stress and lowest linear shrinkage. These findings are consistent with a previous study that concluded that Cention should not be used in self-cure polymerization mode due to its inferior chemical and mechanical properties [52]. For the dual-cured bulk-fill composite Fill-Up!, however, no significant differences between polymerization modes in neither tested static parameter (linear shrinkage, shrinkage stress, or DC) were found, which contrasts with another study that recommends light-curing for both dual-cure bulk-fill materials Cention and Fill-Up to maintain adequate mechanical properties in terms of wear [7].

The polymerization and shrinkage kinetics such as rate and time to achieve maximum rate are important parameters for the chemical and mechanical, and thus clinical, outcomes of a resin composite material [24] and were therefore additionally calculated in the present study. When comparing the kinetic parameters of self-cure materials, it is noticeable that the time to achieve maximum polymerization rate is more than doubled for Cention SC and almost quadrupled for shrinkage stress compared to the self-cure mode of Fill-Up!. As both materials were chemically polymerized, more similar values would have been expected; however, differences might be explained by their different viscosities created by different photo-initiators and in case of Cention alkaline filler systems [10, 53]. It has been shown by several studies that for self-curing materials, a slower polymerization rate can delay the gel point, allowing more resin to flow from the unbonded surface, and can extend the viscous phase, which results in lower shrinkage stress values [47, 54, 55]. This concurs with the results of the present study, which found a tenfold slower shrinkage stress rate for Cention SC compared to Fill-Up! SC. However, the observation that both Cention SC and Fill-Up! SC required significantly more time to achieve the maximum shrinkage stress and degree of conversion rate than their corresponding light-cured counterparts was expected.

Pearson’s correlation analysis considering all experimental groups (light-cure and self-cure) showed a strong linear correlation between linear shrinkage and shrinkage stress. Such a correlation is commonly observed when semi-rigid measuring devices are used [26]. The custom-made stress analyzer employed in the present study belongs to that group of devices, due to its inherent compliance and no external feedback system that would compensate for the displacement [8, 31]. As the amount of linear shrinkage was dominantly dictated by the percentage of resin matrix in the material, the most highly filled composites (Ceram.x Spectra ST (HV), Tetric PowerFill, and Cention) were grouped at the low end of the linear shrinkage and shrinkage stress values, whereas the lower-viscosity materials with lower filler load (SDR flow + , Fill-Up!, and X-flow) were shifted along the correlation line towards higher values of linear shrinkage and shrinkage stress. These results indicate that in a semi-rigid system, flowable composites tend to create higher shrinkage stresses than sculptable composites, despite the latter having higher elastic modulus.

In contrast to the results of the light-cured composites, which were within the commonly expected range for contemporary restorative composites, some parameters of the self-cured dual-cure bulk-fill composites demonstrated highly deviating values. This was especially pronounced for the kinetic parameters (*R*_max_, *t*_Rmax_, *R*_DC,max_, and *t*_R,DC,max_), which differed for an order of magnitude (Fill-Up!) or two orders of magnitude (Cention) from the values measured for the other materials. Unlike these parameters, the “static” parameters measured at the end of the observation period (linear shrinkage, shrinkage stress, and DC) were in a comparatively narrower range (less than half an order of magnitude), regardless of the material type and mode of polymerization. These considerations imply that the self-cured bulk-fill composites underwent a considerably different setting process which may reflect on their polymer network structure. As the polymer network produced by slower polymerization may be more linear with a lower crosslinking density, the effect of self-cure polymerization of the new dual-curing bulk-fill composites on mechanical properties should be further investigated.

The long-term measurements and resulting polymerization kinetics of 4 h of the present study represent one of the innovative aspects of this study, as many studies on DC or shrinkage behavior only consider the first few minutes of polymerization [28, 36, 47, 54, 56]. However, it must generally be mentioned as possible limitation of the present study that the viscosity of the materials under investigation was not measured and therefore classifications are based on the information given by the manufacturers. Since only selected materials could be investigated, a general transferability of the results of the present study to other materials is not possible. Furthermore, it would have been more clinically relevant to test the investigated bulk-fill composite materials in their full material thickness. However, a general specimen thickness of 1.5 mm has been chosen in the current work for standardization purposes for all tested parameters and materials including the dual-cure composite materials.

## Conclusions

It can be concluded that the tested parameters of the composites with their different polymerization modes proved to be highly material dependent, and the higher heterogeneity in material behavior compared to what is commonly observed in light-curing-only restorative materials aggravates the predictability of their effects on clinically relevant parameters.

## Data Availability

Data are available from the authors upon request.
